# SOCIAL FRAILTY AMONG COMMUNITY-DWELLING OLDER ADULTS: A SCOPING REVIEW

**DOI:** 10.1093/geroni/igae098.0686

**Published:** 2024-12-31

**Authors:** Priscilla Carmiol-Rodriguez, Meshack Otewa

**Affiliations:** University of Washington, Washington, United States; University of Washington, Seattle, Washington, United States

## Abstract

The aim of this review is to explore existing evidence concerning social frailty among older adults living in communities, encompassing definitions, protective and risk factors, health outcomes, and interventions. Social frailty, characterized by the lack of social resources and support, intersects with physical and psychological frailty, posing a substantial hazard to successful aging and escalating the likelihood of adverse health consequences. Following PRISMA-ScR guidelines, a systematic search was conducted in MEDLINE, CINAHL, and Web of Science. Inclusion criteria encompassed full-length articles from database journals published in English from October 2016 to July 2023, focusing on community-dwelling adults aged 65 and above, primarily emphasizing social frailty or its interaction with other frailty dimensions. Editorials, gray literature, and white papers were excluded. Thirty-two articles were identified, predominantly from China, Japan, and South Korea. Bunt and Makizako´s definitions (2017; 2018) of social frailty were mainly used in the papers. Social frailty is preceded by conditions such as hearing impairment, metabolic risk factors, and heart disease. It was correlated with depressive symptoms, disability, reduced life satisfaction, and heightened risk of Alzheimer’s disease and physical frailty. Interventions targeting exercise, digital literacy, and technology-assisted screening were proposed to alleviate social frailty’s impact. Addressing social frailty is crucial for enhancing the well-being of older adults in communities, as it predicts a spectrum of adverse health outcomes, impacting both the physical and psychological dimensions of their lives. Nonetheless, further research is vital to refine interventions for preventing and reversing social frailty in this demographic.

